# AG1478 Elicits a Novel Anti-Influenza Function via an EGFR-Independent, GBF1-Dependent Pathway

**DOI:** 10.3390/ijms23105557

**Published:** 2022-05-16

**Authors:** Xu Zhou, Lingxiang Zhu, Cheryl Bondy, Jun Wang, Qianwen Luo, Yin Chen

**Affiliations:** 1 Department of Pharmacology and Toxicology, School of Pharmacy, University of Arizona, Tucson, AZ 85721, USA; zhou@pharmacy.arizona.edu (X.Z.); zhu@pharmacy.arizona.edu (L.Z.); cbondy@email.arizona.edu (C.B.); junwang@pharmacy.arizona.edu (J.W.); 2 Department of Biosystems Engineering, University of Arizona, Tucson, AZ 85721, USA; qianwenluo@email.arizona.edu

**Keywords:** epithelial cells, lung, influenza, virus, GBF1

## Abstract

Current options for preventing or treating influenza are still limited, and new treatments for influenza viral infection are urgently needed. In the present study, we serendipitously found that a small-molecule inhibitor (AG1478), previously used for epidermal growth factor receptor (EGFR) inhibition, demonstrated a potent activity against influenza both in vitro and in vivo. Surprisingly, the antiviral effect of AG1478 was not mediated by its EGFR inhibitory activity, as influenza virus was insensitive to EGFR blockade by other EGFR inhibitors or by siRNA knockdown of EGFR. Its antiviral activity was also interferon independent as demonstrated by a Clustered Regularly Interspaced Short Palindromic Repeats (CRISPR) knockout approach. Instead, AG1478 was found to target the Golgi-specific brefeldin A-resistance guanine nucleotide exchange factor 1 (GBF1)–ADP-ribosylation factor 1 (ARF1) system by reversibly inhibiting GBF1 activity and disrupting its Golgi-cytoplasmic trafficking. Compared to known GBF1 inhibitors, AG1478 demonstrated lower cellular toxicity and better preservation of Golgi structure. Furthermore, GBF1 was found to interact with a specific set of viral proteins including M1, NP, and PA. Additionally, the alternation of GBF1 distribution induced by AG1478 treatment disrupted these interactions. Because targeting host factors, instead of the viral component, imposes a higher barrier for developing resistance, GBF1 modulation may be an effective approach to treat influenza infection.

## 1. Introduction

Influenza infection is among the 10 leading causes of death in the United States and its occasional pandemics kill millions of people around the world. The major preventive measure against influenza infection is through vaccination [1]. However, due to the lengthy manufacturing process, seasonal flu vaccines have to be prepared months before the start of a flu season. Thus, a significant amount of guesswork will be required to estimate potential flu strains that may hit the United States. Furthermore, vaccination is not suitable for everyone. Children younger than 6 months old cannot get a flu vaccine as they are not able to develop a sufficient immune response. Other people that are not suitable for vaccination include those who are allergic to components of the flu vaccine and past and present patients of Guillain–Barré Syndrome (a severe paralyzing illness) [2]. Beside vaccines, antiviral drugs are available to combat flu, and these include M2 channel blockers (e.g., adamantanes including amantadine and rimantadine), neuraminidase inhibitors (e.g., oseltamivir, peramivir, zanamivir), influenza cap dependent endonuclease inhibitor (e.g., baloxavir), and antibodies that neutralize neuraminidase (NA) or haemagglutinin (HA) [3]. However, potential risks of emerging drug resistance prevent their routine use. In fact, amantadines and rimantadines are not recommended for empiric treatment of seasonal influenza as almost all circulating strains demonstrate resistance to these drugs [2]. Although neuraminidase inhibitors are not as prone to viral resistance as adamantanes, viruses containing the oseltamivir resistant mutation (H275Y) rapidly became the predominant seasonal H1N1 strain in 2007–2008 [4]. Thus, new treatments for influenza viral infection are urgently needed. 

The influenza virus belongs to the family of *Orthomyxoviridae*. They can be genetically divided into three groups: influenza A (IAV), B (IBV), and C (ICV) viruses [5]. Among all three groups, IAVs are the most virulent human pathogens and have the potential for severe endemics or pandemics [6]. IAV can be further classified into subtypes based on their two major surface antigens—HA and NA (e.g., H1N1) [5]. A typical influenza viral particle consists of a viral ribonucleoprotein (vRNP) core and a lipid envelope. The vRNP core contains 8 viral RNA segments, and each segment is associated with trimeric RNA polymerase (PB1 PB2 and PA) coated with multiple nucleoproteins (NPs) [5]. The lipid envelope contains two types of glycoproteins (HA and NA) and an M2 ion channel in the form of spikes [7]. Influenza virus enters the host cell by the binding of HAs to sialic acids, the sugars that are attached to a number of cellular membrane proteins [8,9]. C-type lectin can also act as a secondary or co-receptor for influenza infection in macrophages [10]. After viral internalization by the receptor-mediated endocytosis, low endosomal pH activates the M2 proton channel, and the increased proton influx facilitates dissociation of vRNPs from M1 proteins and their subsequent release into the cytoplasm. The unique part of the influenza life cycle is its nucleocytoplasmic trafficking. In the absence of M1 binding, vRNPs are transported into the nucleus to initiate genome replication and mRNA transcription. Viral mRNAs are transported back into the cytoplasm along with cellular mRNAs for viral protein translation, while the newly assembled vRNPs are exported to the cytosol with the assistance of M1 and NEP proteins [5]. Eventually, these vRNPs and other viral proteins are concentrated underneath the cell membrane domains that are already enriched with viral surface proteins such as HA, NA, and M2, and then bud out of the cell surface and cleaved off by NA [5]. 

Airway epithelium is the first line of defense against respiratory influenza infection. Besides their passive role as a physical barrier, epithelial cells actively produce mucins, surfactants, interferons, and proinflammatory cytokines to direct innate and adaptive immune responses to viral infection [11]. Human epidermal growth factor receptor (EGFR)-mediated cellular signaling is critical to maintain epithelial homeostasis. However, its function in influenza infection remains unclear. In one study, EGFR was found to promote IAV internalization by host cells [12]. In the second study, however, EGFR was demonstrated not to affect viral internalization, but instead to facilitate IAV infection by repressing IRF1-dependent interferon (IFN) production [13]. Thus, EGFR was implied as an IAV promoting protein in both studies but via different mechanisms. Our initial objective is to sort out these discrepancies by investigating the mechanistic link between influenza infection and the EGFR signaling pathway. Interestingly, and also surprisingly, EGFR *per se* did not have any effect on influenza infection in our study; rather, the off-target effect of an EGFR inhibitor, AG1478, led to an exciting discovery of a novel anti-influenza pathway.

## 2. Results

### 2.1. A Small-Molecule EGFR Inhibitor, AG1478, Inhibited IAV Production Independent of EGFR Blockade

**Figure 1:**
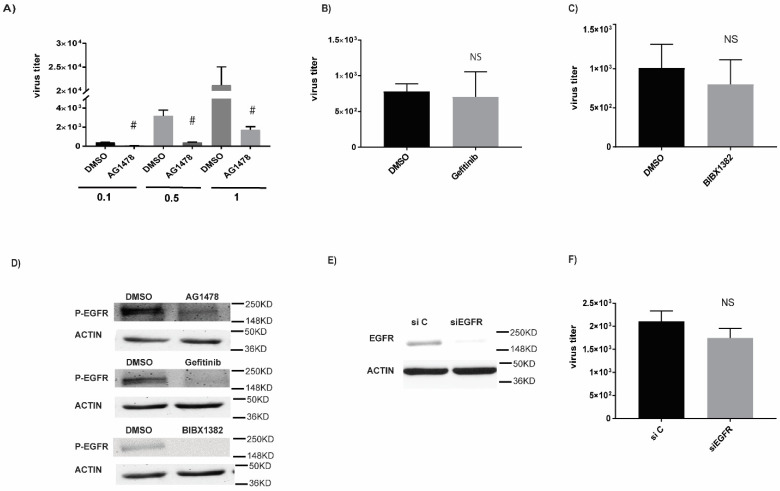
Anti-influenza activity of AG1478 was EGFR independent. Anti-influenza activity of AG1478 was EGFR independent. (**A**) Beas2b cells were treated with 2 μM AG1478 and A/WSN/33 viruses at different MOI (MOI = 0.1, 0.5, 1). Media were collected at 24 h and used for the plaque assay. The assay was carried out with MDCK cells. The same experiments (MOI = 0.1) were also performed with (**B**) 10 μM Gefitinib or (**C**) 5 μM BIBX1382. (**D**) p-EGFR expression was quantified by Western blot analysis. ACTIN was used as a loading control. This is a representative image from three independent repeats. (**E**) siRNA knockdown of EGFR (siEGFR) vs. control siRNA with a random sequence (siC) were analyzed by Western blot analysis. (**F**) Viral titers were analyzed by PFU assay from the cells transfected with siEGFR or siC. For viral titer, data shown are PFU/mL, mean ± SEM. *n* = 4, #: *p* < 0.05. NS: not significant.

To understand the effect of EGFR on IAV production, we initially screened various commercially available EGFR inhibitors (i.e., Gefitinib, BIBX1382, and AG1478) for their antiviral effects. Gefitinib is an FDA-approved TKI for lung cancer. BIBX1382 and AG1478 were discontinued for clinical trials and primarily used as chemical probes for studying EGFR function in the laboratory setting. To our surprise, only AG1478 demonstrated a potent antiviral effect in three different MOIs ranging from a repression by 8-fold at MOI = 0.1 to 21-fold at MOI = 1 (Figure 1A). We also checked a time course of AG1478 activity. At MOI = 0.1, daily dosing of AG1478 demonstrated a long-term anti-IAV activity up to at least 72 h post infection (Supplemental Figure S1). In contrast, neither of the other two EGFR inhibitors (Figure 1B,C) had any effect on IAV production. The lack of antiviral activity was certainly not due to the lack of EGFR inhibition as all three inhibitors significantly repressed EGFR autophosphorylation, an indicator of EGFR activation (Figure 1D). To verify this finding, we knocked down EGFR using EGFR-specific siRNA (siEGFR) (Figure 1E). siEGFR had no significant effect on IAV production as compared to the control siRNA (siC) (Figure 1F). Thus, EGFR inhibition or knockdown appeared to have no antiviral effect.

### 2.2. AG1478 Had a Broad-Spectrum Antiviral Activity across Different Cell Models Independent of IFN

**Figure 2:**
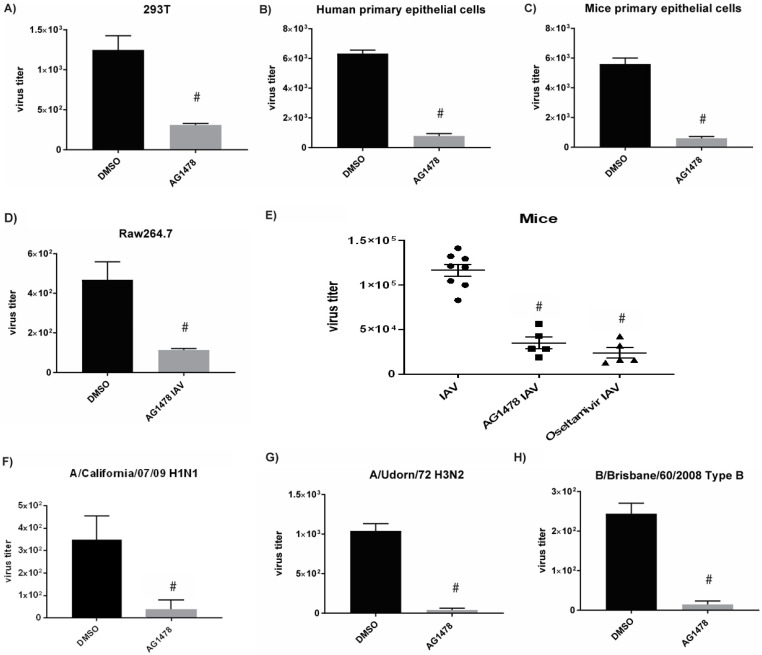
AG1478 had a broad-spectrum antiviral activity across different cell models. Cells were infected with A/WSN/33 viruses and anti-influenza activities of AG1478 were tested in (**A**) 293T cells, (**B**) human primary epithelial cells, (**C**) mice primary epithelial cells, and (**D**) Raw 264.7 cells, *n* = 4. (**E**) C57BL/6J mice were treated with 20 mg/kg AG1478 (*n* = 5) or 20 mg/kg Oseltamivir (*n* = 5) or vehicle control (*n* = 8). Then, lung viral titers (PFU/g) were determined. Beas2B cells were infected with (**F**) A/California/07/09 H1N1, (**G**) A/Udorn/72 H3N2, and (**H**) B/Brisbane/60/2008 Type B under the treatment of 2μM AG1478. *n* = 4. Data shown are PFU/mL, mean ± SEM. #: *p* < 0.05.

To further characterize this interesting finding, we tested antiviral effects of AG1478 in different cell models. AG1478 demonstrated potent antiviral activity against H1N1 (A/WSN/33) in 293T cells, an immortalized kidney epithelial cell line (Figure 2A), in human primary airway epithelial cells (Figure 2B), in mouse primary airway epithelial cells (Figure 2C), and in Raw264.7, a mouse macrophage cell line (Figure 2D). These results extended our previous finding in an immortalized airway epithelial cell model to other useful models of human and mouse. Importantly, antiviral effects of AG1478 were preserved in primary cells of mouse or human, implying that this in vitro finding can be translated to animal models or human subjects. Indeed, in a pilot study using an animal model of IAV infection, AG1478 treatment demonstrated a significant antiviral activity to reduce lung viral titer by ~3.4 times as compared to the mice treated with a vehicle control (Figure 2E). In comparison, an FDA-approved anti-influenza drug oseltamivir reduced lung viral titer by ~4.8 times (Figure 2E). Thus, AG1478 was also able to elicit an anti-influenza activity in vivo. Additionally, in the Beas2b cells, AG1478 effectively repressed a different IAV-H1N1 (A/California/07/09) (Figure 2F), IAV-H3N2 (A/Udorn/72) (Figure 2G), and a type B Influenza virus or IBV (B/Brisbane/60/2008) (Figure 2H), suggesting that the antiviral activity of AG1478 was not strain-dependent.

**Figure 3:**
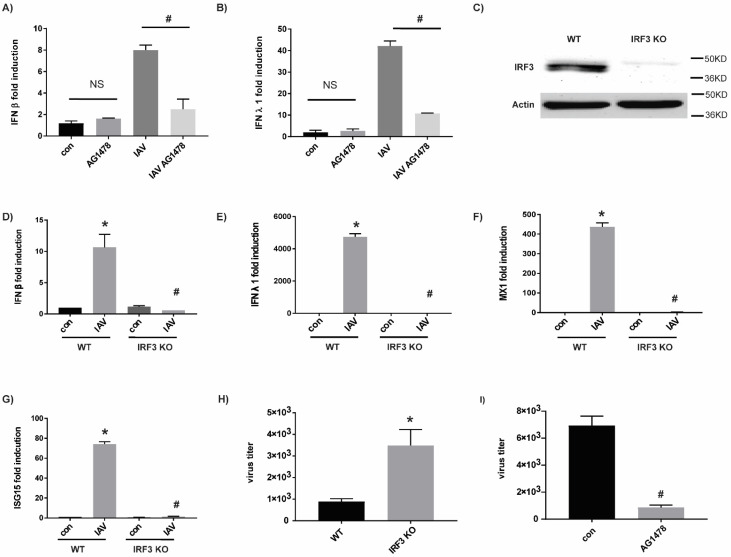
Anti-influenza activity of AG1478 was IFN independent. Beas2b cells were treated with 2 μM AG1478 and infected with A/WSN/1933, and gene expressions of (**A**) IFN β and (**B**) IFN λ1 were quantified by using real-time PCR. (**C**) IRF3 was knocked out by CRISPR. IRF3 protein expression was detected in wild-type cells (control), but not in the knockout cells (IRF3-KO). ACTIN was the loading control. This is a representative image from three independent repeats. In the wild-type or IRF3-KO cells, (**D**) IFN β, (**E**) IFN λ1, (**F**) MX1, and (**G**) ISG15 gene expressions were measured by using real-time PCR. (**H**) Wild-type and IRF3-KO cells were infected with A/WSN/33 influenza viruses for 24 h, and viral titers were quantified by plaque assay. (**I**) IRF3-KO cells were treated with 2 µM AG1478 and infected with A/WSN/33 influenza virus (MOI 0.1), and viral titers were quantified by plaque assay. Data shown are mean ± SEM. *n* = 3, * and #: *p* < 0.05. NS: not significant.

Because the IFN system is indispensable for cellular antiviral defense, we tested if the antiviral activity of AG1478 was mediated through IFN. We found that AG1478 alone did not induce any expression of type I IFN (IFNβ) (Figure 3A) or type III IFN (IFNλ1) (Figure 3B). Notably, type II IFN (IFNγ) was not expressed by epithelial cells (data not shown). Interestingly, AG1478 repressed IAV-induced IFN (Figure 3A,B), suggesting the antiviral activity of AG1478 may be independent of the IFN pathway. To confirm this finding, we specifically knocked out IRF3, the common upstream regulator of type I and III IFN pathways [14,15] by CRISPR approach. The lack of IRF3 protein in IRF3-KO cells was confirmed by a Western blot analysis (Figure 3C). The lack of IRF3 significantly repressed IAV-induced IFN (IFNβ or IFNλ1) (Figure 3D,E) and downstream IFN-regulated genes (MX1 or ISG15) (Figure 3F,G) as compared to wild-type cells. Consistently, the lack of the IFN pathway significantly elevated IAV production (Figure 3H). These data confirm that IRF3-KO cells were completely devoid of any IFN system. Interestingly, AG1478 still preserved its antiviral activity in these IRF3-KO cells (Figure 3I). Thus, the antiviral activity of AG1478 was not mediated through the IFN system. 

### 2.3. The Target of AG1478 Is GBF1

**Figure 4:**
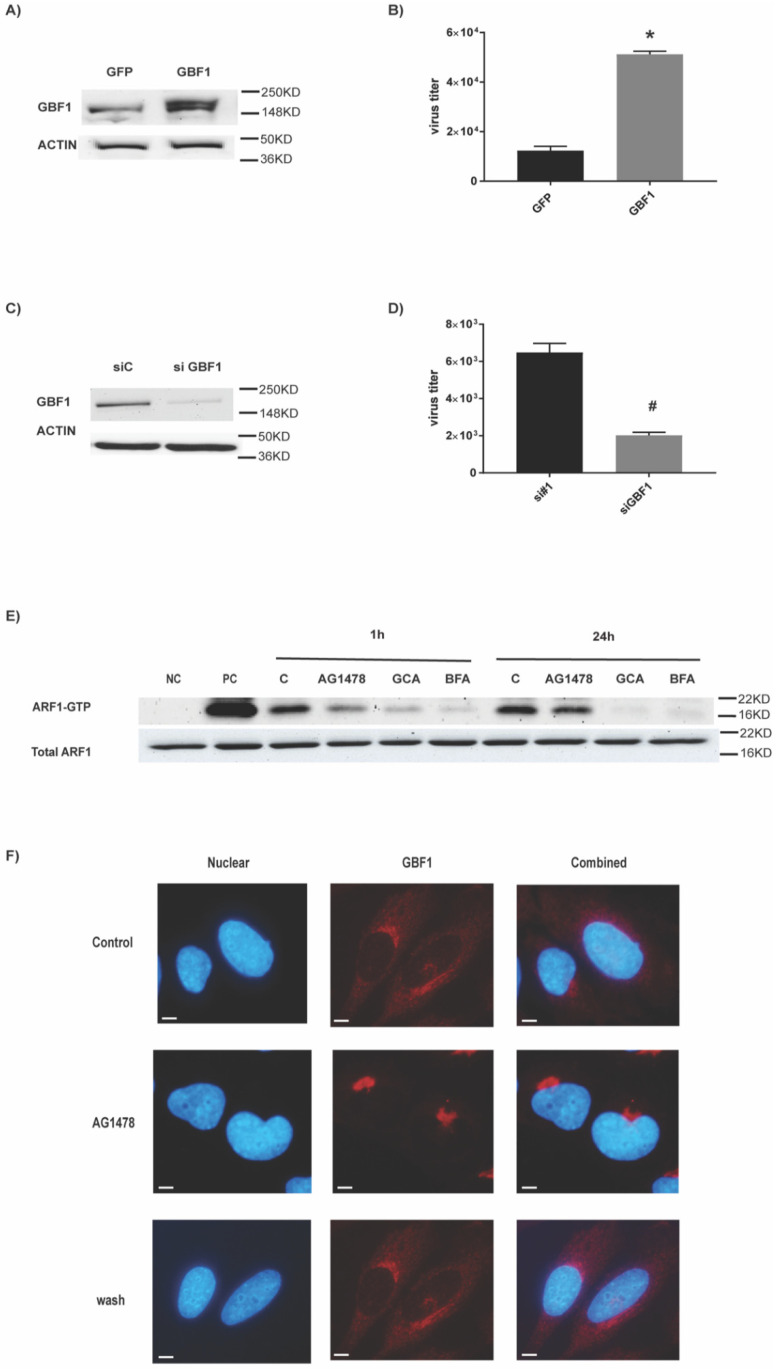
AG1478 targets GBF1. (**A**) Cells were transfected with either GBF1-GFP (overexpression) or GFP plasmid alone (control). GBF1 protein expression was detected by Western blot analysis. ACTIN was used as a loading control. (**B**) Viral titers were quantified by plaque assay in GBF1 overexpressed or GFP expressed cells. Triplicates were used for each experiment and experiments were repeated at least three times. Data shown are mean ± SEM. *n* = 4, * and #: *p* < 0.05. (**C**) GBF1 siRNA (siGBF1) was used to knock down GBF1. Cells transfected with siRNA with a random sequence (siC) were used as control. (**D**) Viral titers were quantified by plaque assay in siGBF1 or siC-transfected cells. (**E**) Cells were treated with AG1478, BFA, and GCA, and ARF1-GTP was pulled down. Total ARF1 protein was used as a loading control. This is a representative image from three independent repeats. (**F**) Cells were treated with AG1478 for 24 h, and then they were fixed and stained for GBF1 (Red). AG1478-containing media were removed from some of the wells and replaced with new media without AG1478 at 1 h before fixation (labeled as wash). DAPI (blue) was used for the nuclear staining. Images were taken under 63× magnification. Scale bar: 5 µm.

Off-target effects of AG1478 were demonstrated previously [16,17,18]. GBF1, a potential AG1478 cellular target [16], was also identified as one of the 91 host factors that affect IAV production [19]. Thus, we tested the hypothesis that the antiviral effect of AG1478 was mediated by GBF1. Indeed, compared with the overexpression of an irrelevant protein-GFP (control), GBF1 overexpression (Figure 4A) significantly enhanced IAV production (Figure 4B). Reciprocally, GBF1 knockdown by a siRNA against GBF1 (siGBF1) (Figure 4C) repressed IAV production (Figure 4D). Thus, GBF1 was required for IAV production.

GBF1 is the guanine nucleotide exchange factor (GEF) that catalyzes GDP/GTP exchange of ARFs from their inactive GDP-bound (ARF1-GDP) to active GTP-bound (ARF1-GTP) form [20,21,22]. ARF1 is one of the downstream substrates of GBF1, and the amount of ARF1-GTP complex was a direct indicator of GBF1 activity. In our study, AG1478 treatment significantly reduced the amount of ARF1-GTP complex (Figure 4E), confirming the notion that AG1478 repressed GEF activity of GBF1. Additionally, the inhibitory kinetics of AG1478 were different from two established GBF1 inhibitors (i.e., GCA and BFA) [23]. GEF activity of GBF1 was mostly restored within 24 h under the treatment of AG1478, but not GCA or BFA (Figure 4E). GBF1 has also been shown to shuffle between cytoplasm and Golgi [22]. We found that AG1478 treatment concentrated GBF1 in the Golgi at the expense of its cytoplasmic pool (Figure 4F). There was very little cytoplasmic GBF1 after 24 h treatment of AG1478. Although this effect lasted longer than the inhibition of GEF activity, it was reversible as AG1478 washout quickly restored cytoplasmic GBF1 within 1 h (Figure 4F). Nonetheless, daily dosing of AG1478 demonstrated persistent antiviral activity as shown in Supplemental Figure S1. 

### 2.4. AG1478 Had a Superior Safety Profile as Compared to GCA and BFA

**Figure 5:**
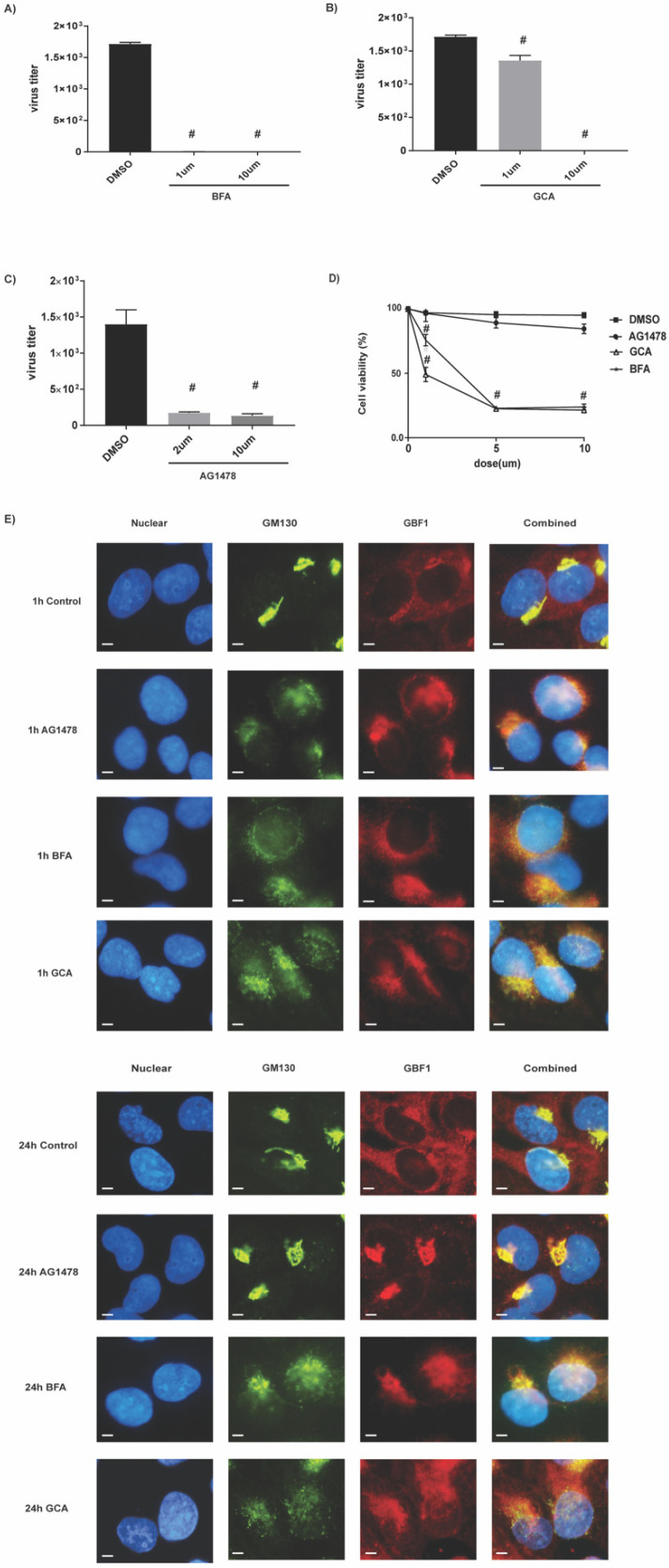
AG1478 targeted on GBF1. Beas2B cells were treated with (**A**) BFA at 1 and 10 μM, (**B**) GCA at 1 and 10 μM, and (**C**) AG1478 at 2 and 10 μM and infected with A/WSN/33 influenza virus. (**D**) MTT assay was used to test cell viability after 24 h treatments of BFA, GCA, and AG1478 at indicated doses. Quadruplicates were used for each experiment and experiments were repeated at least three times. Data shown are mean ± SEM. *n* = 4, #: *p* < 0.05. (**E**) The 1 and 24 h treatment of BFA, GCA, and AG1478. GM130 (green, a Golgi marker) and GBF1 (red) were measured by immunofluorescence. DAPI (blue) was used for the nuclear staining. Co-staining was shown in a combined image. Images were taken under 63× magnification. Scale bar: 5 μm. All were representative images from three independent repeats.

As two existing GBF1 inhibitors (BFA and GCA) demonstrated different inhibitory characteristics as compared to AG1478 (Figure 4E), we decided to compare these three compounds for their antiviral activities. All compounds demonstrated decent antiviral activity (Figure 5A–C). However, AG1478 induced very little cellular toxicity across the entire dose range (1, 5, 10 µM) as demonstrated by a cell viability assay. In contrast, significant toxicity was observed for BFA and GCA even at 1 μM (Figure 5D). For GCA, its viral inhibition appeared to be mostly caused by cell death (Figure 5B). 

As GBF1 activity is essential to maintain Golgi integrity, we tested how these three chemicals affected Golgi by fluorescence microscopy. In contrast to BFA and GCA that induced persistent Golgi dispersion, AG1478 only caused transient Golgi dispersion and it was fully recovered within 24 h (Figure 5E). Combining Figure 4E and Figure 5, the antiviral activities of these three GBF1 inhibitors appeared not to be proportional to their effects on Golgi dispersion as well as to their cellular toxicity. Nevertheless, AG1478 demonstrated a low cellular toxicity, a mild effect on Golgi structure, and a potent antiviral activity. Thus, AG1478 is clearly the top candidate for antiviral drug development among these three chemicals.

### 2.5. GBF1 Interacted with Selected Viral Proteins

**Figure 6:**
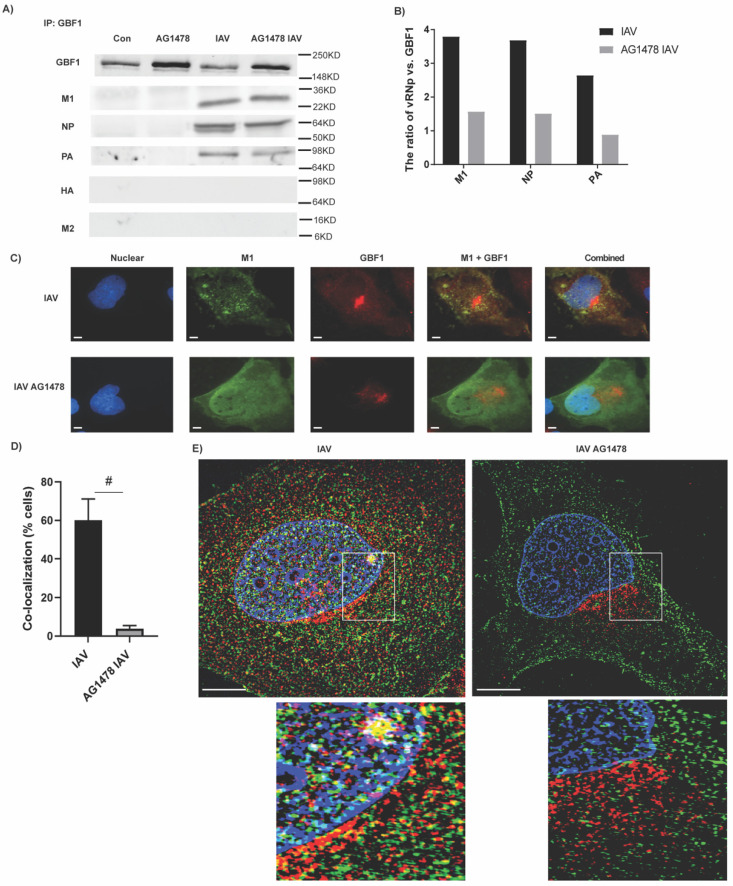
GBF1 interacted with viral proteins. (**A**) Beas2b cells were treated with 2 μM AG1478 and infected with A/WSN/1933 for 24 h. GBF1 antibody was used for immunoprecipitation. The precipitants were analyzed for GBF1, NP, M1, PA, HA, and M2. (**B**) Quantification of the ratio of IAV protein over GBF1 in (**A**). These were the representation from three independent repeats. (**C**) GBF1 (red) and M1 (green) were co-localized (yellow) in the absence of AG1478, but completely dissociated in the presence of AG1478. DAPI (blue) was used for the nuclear staining. Images were taken under 63× magnification. Scale bar: 5 μm. (**D**) Quantification of percentage of cells having co-localization signals. Data shown are mean ± SEM. *n* = 10, #: *p* < 0.05. (**E**) Super-resolution microscopy under 100× magnification. Scale bar: 5 μm. The co-localization of GBF1 (red) and M1 (green) was shown by yellowish colored dots in the enlarged images. All were representative images from three independent repeats.

To further understand the mechanistic basis of the antiviral effect of AG1478, we tested its effect on viral transcription by real-time PCR measurements of individual viral RNAs (Supplemental Figure S2A,B), on viral protein translation by Western blot analysis (Supplemental Figure S2C), and on viral genome replication by a minigenome assay (Supplemental Figure S2D). Surprisingly, AG1478 did not affect any of these processes. By immunoprecipitation, GBF1 was found to interact with selected viral proteins: M1, NP, and PA, but not HA and M2 (Figure 6A). All of these interacting proteins were responsible for nucleoplasmic trafficking of vRNP [5]. Interestingly, a greater amount of GBF1 was precipitated when the cells were treated with AG1478 as compared with the control (Figure 6A), even though equal amounts of proteins from each sample were loaded. Although the absolute amount of each precipitated vRNP protein was not significantly changed by the treatment of AG1478 (Figure 6A), their ratio to precipitated GBF1 was decreased by greater than 2-fold when treated with AG1478 (Figure 6B), suggesting that the interaction between GBF1 and vRNP protein was indeed impaired by the treatment of AG1478. As a control, we also analyzed input proteins before IP analysis. As shown in Supplemental Figure S3, total GBF1 proteins were increased by AG1478 treatment irrespective of viral infection, which was consistent with IP data (Figure 6A) and suggested that AG1478 may affect GBF1 protein production. In addition, both HA and M2 were present in the input proteins (Supplemental Figure S3) but absent from immunoprecipitants (Figure 6A). Because AG1478 altered the cellular distribution of GBF1 from Golgi-cytoplasmic to Golgi-centric (Figure 4F and Figure 5E), we reasoned that the immunoprecipitation on a whole cell lysate might not be sufficient to capture the overall effect of AG1478. Thus, we performed an immune-fluorescence co-staining using the antibodies against viral M1 protein and GBF1. Significant co-localization of M1 and GBF1, as demonstrated by yellowish co-staining pixels, was indeed observed in the absence of AG1478. However, when the cells were treated with AG1478, M1 and GBF1 were completely dissociated (Figure 6C). Quantitively, in the cells without AG1478, greater than 60% of cells showed co-localization of M1 and GBF1. In contrast, less than 4% of AG1478-treated cells showed co-localization (Figure 6D). This dramatic effect by AG1478 was further confirmed by using super-resolution microscopy (Figure 6E). In the enlarged images, a large number of yellow-colored spots were readily visible in IAV-infected cells but were almost completely absent in AG1478-treated cells.

## 3. Discussion

Because of its ability to modulate multiple ARFs and to control vesicle and lipid trafficking [24,25], GBF1 is an ideal host factor to be hijacked by positive-strand RNA viruses (PSRVs) to establish specialized membranous compartments for efficient RNA replication and for successful evasion of cellular pattern recognition receptors and innate defense [26]. GBF1 has been reported to play a key function for the replication of a number of PSRVs including *Coronaviridae* [27,28], *Flaviviridae* [24,29,30,31,32], *Picornaviridae* [23,33,34,35,36,37], and *Togaviridae* [38].

However, the role of GBF1 has rarely been studied on non-PSRVs. The only two available references in this area are about the high-throughput screening of cellular responses to vesicular stomatitis virus [39] or of protein interactome by IAV proteins [19]. GBF1 was identified as one of the 91 host factors that significantly affect IAV genome production, but no follow-up analysis was performed [19]. To our best knowledge, the present study is perhaps the only mechanistic study of GBF1 function on a non-PSRV. Different from its role in PSRVs, GBF1 did not affect IAV replication. This is consistent with the spatial separation of IAV replication (nucleus) and GBF1 protein (cytosol/Golgi). Instead, GBF1 was found to interact with several viral proteins including M1, NP, and PA, all of which are important for vRNP assembly and trafficking. Because vRNP is believed to be transported to the budding location via membrane-bound vesicles, IAV may hijack GBF1 for this task. Indeed, GBF1 was found in our study to be co-localized with M1 protein, a master regulator of intracellular vRNP trafficking. The only other well-characterized GBF1-interacting viral protein is the non-structural protein 3A in poliovirus or coxsackievirus. 3A is a small hydrophobic membrane protein and was shown to inhibit GBF1 [40,41], thereby interfering with ARF1-GBF1-mediated endoplasmic reticulum-to-Golgi complex transport. For these PSRVs, GBF1 was proposed to redirected to recruit other supportive host factor (e.g., PI4Kα) for viral replication [42]. In our study, although the nature of the interaction between GBF1 and M1 is not clear, ectopic GBF1 overexpression was found to enhance and GBF1 knockdown was found to inhibit viral production. The inhibition of viral production by AG1478-elicited alteration of GBF1 localization further supports the favorable role of GBF1 in facilitating viral production.

AG1478 itself has also demonstrated antiviral activities against Encephalomyocarditis Virus (EMCV) [18], Hepatitis C Virus (HCV) [18], Marburg virus [43], Lassa virus [43], and Ebola virus [43]. For EMCV and HCV, Phosphatidylinositol 4-Kinase IIIα (PI4Kα) was found to be the target of AG1478 based on its structural similarity to a known PI4Kα inhibitor—AL-9. For Marburg, Lassa, and Ebola viruses, >12.5 μM of AG1478 was required to elicit antiviral activity [43]. However, no precise mode of action was elucidated. Since AG1478 completely inhibited EGFR activity at low μM range, its EGFR inhibitory activity was not likely responsible for the antiviral activity against these hemorrhagic fever viruses. In this study, we are the first to link the anti-influenza activity of AG1478 with GBF1, another non-EGFR target. Thus, although AG1478 is a well-recognized EGFR inhibitor, its antiviral activity appears to be EGFR-independent.

The effect of AG1478 on GBF1 was first reported by Pan H et al. in an image-based phenotypic screen to identify the small molecule regulator of intracellular traffic [16]. In their study using a 1 h short-term treatment protocol [16], AG1478 elicited its effect on Golgi dispersion through the conserved catalytic Sec7 domain of GBF1 [16]. The Sec7 domain has also been demonstrated to be responsible for the interaction between GBF1 and two other inhibitors—GCA and BFA. In our study, however, Golgi dispersion appeared to be transient and was fully recovered within 24 h. This effect was different from GCA and BFA, as the treatment of any of these two inhibitors rendered Golgi dispersion persistent. This discrepancy suggests that GBF1 may interact with AG1478 differently from GCA/BFA. Of note, both catalytic Sec7 [42] and non-catalytic N-terminal [33,44] domains of GBF1 were required for polioviral replication. Thus, the specific domain(s) of GBF1 that interacts with AG1478 will warrant further study.

Targeting host factors, instead of viral proteins, has been an alternative strategy for antiviral therapy holding the promise of producing broad-spectrum antivirals and at the same time raising the barrier for the emergence of drug resistant mutants. One major concern for targeting host factors is their potential cellular toxicity, as the candidate host factor may also play important roles in host cell homeostasis. In our study, several unique characteristics about the interaction between AG1478 and GBF1 suggest that the future therapy based on this finding may be safe. First, the inhibition of GBF1 by AG1478 only caused transient Golgi dispersion and it was fully recovered within 24 h. In contrast, two other GBF1 inhibitors, GCA and BFA, caused more extended dispersion of Golgi apparatus. This is also consistent with the kinetics of ARF1 inhibition. As a result, AG1478 treatment did not cause significant cellular toxicity compared to GCA or BFA. Second, influenza appeared to hijack a significant portion of GBF1 for its own protein transport in the cytoplasm. Thus, the effect of AG1478 on the Golgi-cytoplasmic trafficking of GBF1 may preferentially impact viral production since the normal cellular function is kept at a much lower level under this pathological condition. It will then be very interesting to find out whether the mode of action of GBF1 in the IAV-related vesicle trafficking is different from the normal. Third, the normal function of GBF1 could be restored by washing out AG1478. This is significant because the GBF1 system appears to be intact after inhibition and the future dosing can be carefully formulated to target certain windows of the infection while minimizing unwanted side effects. Although this strategy holds the promise to overcome drug resistance, we did not test if IAV can produce a drug-resistant strain in the presence AG1478. A BFA-resistant mutant of polio virus was selected previously. However, this resistant phenotype was cell-type-specific (i.e., BFA-resistant phenotype is restrained in the cells that were used for selection, but not in other cell types), and the replication of the mutant was slow and prone to cellular innate defense such as the IFN system. Thus, even though mutants bypassing host-factor-targeting drugs can emerge, they appear to be less adaptive and have trade-off defects in the evasion of immune surveillance, suggesting that these mutants can be either cleared automatically by the host immunity or be treated by an immune modulator (e.g., CpG etc.) to boost host immune response.

In summary, through a serendipitous finding, we discovered a potent anti-influenza drug candidate—AG1478. Its antiviral activity is IFN independent and is mediated by targeting GBF1, a regulator of intracellular trafficking. GBF1 appears to be hijacked by IAV for its own transport via the interaction between GBF1 and M1 as well as its associated vRNP. The treatment of AG1478 disrupted this interaction and potentially impaired vRNP transport leading to markedly decreased IAV production. Further development on this candidate will lead to novel anti-influenza therapy.

## 4. Materials and Methods 

### 4.1. Cell Culture and Virus 

Beas2b cells were obtained from American Type Culture Collection (ATCC, Manassas, VA, USA), and cultivated on a regular tissue culture dish in RPMI media plus 10% fetal bovine serum (FBS). HELA, 293T, and Raw264.7 cells were also obtained from American Type Culture Collection (ATCC, Manassas, VA, USA), and cultivated on the regular tissue dish in DMEM plus 10% FBS. Human bronchial tissues were purchased from National Disease Research Interchange. Primary human bronchial epithelial cells were isolated and cultivated under an immersed condition as described previously in a Ham’s F12:Dulbecco’s modified Eagle’s medium (1:1) supplemented with eight factors, including: insulin (5 mg/mL), transferrin (5 mg/mL), epidermal growth factor (10 ng/mL), dexamethasone (0.1 mM), cholera toxin (10 ng/mL), bovine hypothalamus extract (15 mg/mL), bovine serum albumin (0.5 mg/mL), and all-trans-retinoic acid (30 nM) [45]. Ethics statement: human bronchial tissues were purchased from National Disease Research Interchange. These tissues were all de-identified discarded samples, and no individual identity could be ascertained. Primary mice tracheobronchial epithelial cells were isolated from C57BL/6J mice and cultivated as described previously [46]. MDCK cells were obtained from Dr. Jun Wang’s lab and cultivated on a regular tissue culture dish in DMEM media plus 10% fetal bovine serum (FBS). MDCK cells overexpressing ST6Gal I were obtained from Dr. Jun Wang’s lab and were maintained in the presence of 7.5 μg/mL of puromycin, except when they were used for viral infection [47]. A/WSN/33(H1N1), A/California/07/09 (H1N1), A/Udorn/72(H3N2), and B/Brisbane/60/2008 were generous gifts from Dr. Jun Wang. Virus stocks were amplified in MDCK cells in the presence of 2 μg/mL N-Acetyl trypsin for multi-cycle replications. After two days of infections, the culture medium was harvested by centrifugation and viral titers were determined by plaque assay [48].

### 4.2. Plaque Assay

Viral samples from the supernatant of infected cells were 2-fold serially diluted with DMEM plus 0.5% BSA. Additionally, the serially diluted medium was used to inoculate 100% confluent monolayer MDCK for 1 h at 4 °C, and then moved to 37 °C for 1 h. Equal volumes of 2X DMEM and 2.4% Avicel were mixed, and then N-acetylated trypsin was added. The final concentration was 1.2% Avicel, 1× DMEM final concentration, and 2 µL/mL N acetylated trypsin. After incubation for 48 h followed by Crystal violet staining, plaque forming units (PFUs) were quantified [47,48].

### 4.3. A Mouse Model of Influenza Infection 

Six-week-old C57BL/6J mice were treated with 20 mg/kg AG1478 via laryngeal aspiration 3 h before IAV infection (1 × 10^4^ A/WSN/33 per mouse). The mice were given a second dose of AG1478 on the 2nd day and tissues were harvested on the 3rd day. Mouse lungs were homogenized followed by centrifugation. Supernatants were subject to plaque assay as described above. The control mice were given a vehicle control (DMSO). In a separate experiment, oseltamivir (Sigma-Aldrich, St. Louise, MO, USA) was used as a positive control and given to the mice at 20 mg/kg via the same laryngeal aspiration following the same protocol as AG1478. Ethics Statement: Mice used in the present study were covered under the animal protocol (#13-479) that was approved by the Institution Animal Care and Use Committee (IACUC) of the University of Arizona. Mice used in this study were housed in a barrier facility at the University of Arizona. The facility is accredited by the American Association for Accreditation of Laboratory Animal Care (AAALAC) and meets the requirements of the law and NIH regulations. All mice are routinely screened for a panel of pathogens and are cared for by certified animal care professionals. All the animal handling and experimental procedures adhere strictly to the general guidelines of the Animal Welfare Act (AWA), AAALAC and IACUC.

### 4.4. Inhibitors and Treatments

Cells were pretreated for 1 h with inhibitors before the infection. Then, they were washed and incubated with viruses at different multiplicities of infection (MOI) as indicated for 1 h. The infection was stopped by washing cells repeatedly with PBS to ensure that no residue virus was left. Finally, cells were further incubated with culture media containing inhibitors for various time points as indicated. 

### 4.5. RNA Extraction, cDNA Synthesis, and Real-Time Quantitative PCR (qPCR)

Total RNA was extracted from cells using Trizol reagent (Invitrogen, Carlsbad, CA, USA). cDNA was prepared from 2 mg of total RNA and was then further diluted to 100 μL with water for the following procedures. Two microliters of diluted cDNA were analyzed using SYBR Green PCR Master Mix by a Veriti^®^ Thermal Cycler (Thermo Fisher, Grand Island, NY, USA). Primers were used at 0.2 mM. The primer was designed by us using the Primer 3 software. The relative mRNA amount in each sample was calculated based on the ΔΔCt method using the housekeeping gene β-Actin. Results were calculated as fold induction over control [46]. Primers are listed in Table 1.

### 4.6. Antibodies and Western Blot

Total cellular proteins were collected based on the methods described previously [45]. Antibodies for p-EGFR, EGFR, IRF1, and IRF3 were purchased from Cell Signaling Company (Denver, MA, USA). Antibodies for IAV M1, M2, NP, PA, HA, and NA were purchased from Genetex (Irvine, CA, USA). Antibodies for ARF1 and GBF1 were purchased from Santa Cruz Biotechnology (Dallas, TX, USA). Equal protein loading was confirmed using anti-Actin antibody (Dallas, TX, USA). The experiment was repeated at least three times. 

### 4.7. Gene Knockout Using Clustered Regularly Interspaced Short Palindromic Repeats (CRISPR)

Small guide RNAs (sgRNA) (5′- AGAAGGGTTGCGTTTAGCAG-3′ and 5′-CCCGGGAACATATGCACCAG-3′) were designed to target exon 3 in the human IRF3 gene using the CRISPR gRNA design website at the Broad Institute (https://portals.broadinstitute.org/gpp/public/analysis-tools/sgrna-design (accessed on 5 March 2022)). Complementary oligos for the gRNAs were synthesized by Sigma-Aldrich and cloned into LentiCRISPRv2 (Addgene, Cambridge, MA, USA) based on a protocol by the Zhang lab [49,50]. Plasmids containing sgRNAs were confirmed by sequencing at Genewiz (South Plainfield, NJ, USA). 293T cells were transfected with lentiCRISPRv2 containing sgRNA, psPAX2 (Addgene, Cambridge, MA, USA), and VSVg (Addgene, Cambridge, MA, USA) by Lipo2000 (Invitrogen, Waltham, MA, USA) to produce lentiviral particles containing LentiCRISPR construct. To create knockout cell lines, Beas2b cells were transduced and selected using 2 μg/mL puromycin. IRF3 knockout cell clones were confirmed by Western blot.

### 4.8. Immunofluorescence

Cells were fixed with 2% paraformaldehyde and permeated with Triton X-100. Cellular proteins were determined by immunofluorescence using specific antibodies against GBF1 (BD Biosciences, San Jose, CA, USA), GM130 (Abcam, Cambridge, UK), and IAV M1 (Genetex, Irvine, CA, USA). Alexa 488 and 594 conjugated secondary antibodies (Thermo Fisher, Grand Island, NY, USA) were used to obtain co-stained fluorescence images. DAPI was used to stain the cell nucleus. The images were acquired either by Nikon microscope (Nikon Eclipse Ti Microscope, Tokyo, Japan) or by super-resolution microscopy (ELYRA S.1, Carl Zeiss, Thornwood, NY, USA). Images were acquired by a 63×/1.40 plan-apochromat or a 100×/1.46 alpha plan-APO oil immersion objective lens and post-processed using Zeiss ZEN Black version 2.3 (Carl Zeiss, White Plains, NY, USA) software. All imaging analyses were repeated at least three times, and major features were determined by two independent examiners in a blinded fashion. The degree of colocalization of GBF1 and M1 was determined by randomly sampling 10 fields in each individual sample and counting the percentage of cells showing co-staining. In total, 10 replicates were performed.

### 4.9. Transfection

GBF1 plasmid was a generous gift from Dr. Paul Melancon (University of Alberta, Canada). siRNA sequence [51] was synthesized by Sigma-Aldrich (St. Louis, MO, USA). Plasmid or siRNA were transfected into the cells using the Lipofectamine 2000 (Invitrogen, Waltham, MA, USA) according to the manufacturer’s suggested protocol.

### 4.10. Cell Viability Assay

Cells were treated with various inhibitors for 24 h. Additionally, the cell viability was analyzed by using the CellTiter 96 Non-Radioactive Cell Proliferation Assay Kit (Promega, Madison, MI, USA). The absorbance was measured at 492 nm using a plate reader (Tecan, Männedorf, Switzerland). The percentage of viability was calculated as: treatment/control (DMSO) × 100. Control was designated as 100% viability.

### 4.11. Immunoprecipitation and ARF1 Activity Assay

To test for coimmunoprecipitation, the precipitated proteins were separated by SDS-PAGE and immunoblotted using antibodies against GBF1 (Santa Cruz, CA, USA), IAV M1, NP, and PA (Genetex, Irvine, CA, USA). Active ARF1 pull down and detection kit (Thermofisher, Waltham, MA, USA) was used to quantify the amount of GTP bound ARF1 (ARF-GTP) based on the manufacturer’s protocol. Equal amounts of proteins were loaded for the pull-down.

### 4.12. Statistical Analysis

Experimental groups were compared using a two-sided Student’s *t* test, with significance level set as *p* < 0.05. When data were not distributed normally, significance was assessed with the Wilcoxon matched-pairs signed-ranks test, and *p* < 0.05 was considered to be significant.

## Figures and Tables

**Table 1:** PCR primers.

Gene	Primer
β-Actin	Forward ACTGGAACGGTGAAGGTGACA
	Reverse ATGGCAAGGGACTTCCTGTAAC
IFNβ	Forward ATTGCCTCAAGGACAGGATG
	Reverse GCTGCAGCTGCTTAATCTCC
IFNλ1	Forward GGACGCCTTGGAAGAGTCACT
	Reverse AGAAGCCTCAGGTCCCAATTC
IFNλ2/3	Forward CTGCCACATAGCCCAGTTCA
	Reverse AGAAGCGACTCTTCTAAGGCATCTT
MX1	Forward AGAGAAGGTGAGAAGCTGATCC
	Reverse TTCTTCCAGCTCCTTCTCTCTG
ISG15	Forward GGACCTGACGGTGAAGATGCT
	Reverse ACGCCAATCTTCTGGGTGATCT

## Data Availability

Data is contained within the present article and supplementary material.

## Supplementary Materials

The following are available online at https://www.mdpi.com/article/10.3390/ijms23105557/s1 [52].
