# Supplementary material for: AG1478 Elicits a Novel Anti-Influenza Function via an EGFR-Independent, GBF1-Dependent Pathway
Source: Int J Mol Sci. 2022 May 16;23(10):5557. doi: 10.3390/ijms23105557 (PMC9145774; doi:10.3390/ijms23105557)
Supplement: Supplementary file 1 [file ijms-23-05557-s001.zip › ijms-1704197-supplementary.pdf]

A small-molecule tyrosine kinase inhibitor elicits a novel anti-influenza function via an EGFR-independent, GBF1-dependent pathway

[1]Xu Zhou, [1]Lingxiang Zhu, [1]Cheryl Bondy, [1]Jun Wang, [2]Qianwen Luo and [1]Yin Chen*

[1] Department of Pharmacology and Toxicology, School of Pharmacy, University of Arizona, Tucson, AZ.

[2]Department of Biosystems Engineering, University of Arizona, Tucson, AZ.

* Corresponding author: ychen@pharmacy.arizona.edu; Tel: 520-626-4715; Fax: 520-626-2466

**Supplemental Materials and Methods:**

Minigenome assay (1)

Cells were transfected with pCDNA constructs for influenza A/WSN/33 virus PB1, PB2, and PA (100 ng each) and NP (200 ng), the RNA polymerase II-driven Renilla luciferase reporter pRL-SV40 (Promega) (250 ng), and the influenza virus-specific RNA polymerase I driven firefly luciferase reporter (vRNALuc) (250 ng). The transfection was performed with TransIT-293(Mirus) in OptiMEM (Invitrogen). Twenty-four hours after incubation, cells were harvested, and firefly luciferase and Renilla luciferase expression were determined using the Dual Luciferase Assay Kit (Promega).

**Reference:**

52. Hoffmann H.H, Kunz A., Simon V.A., Palese P., Shaw M.L. Broad-spectrum antiviral that interferes with de novo pyrimidine biosynthesis. *Proc Natl Acad Sci* USA **2011**, *108*, 5777-5782, doi: 10.1073/pnas.1101143108

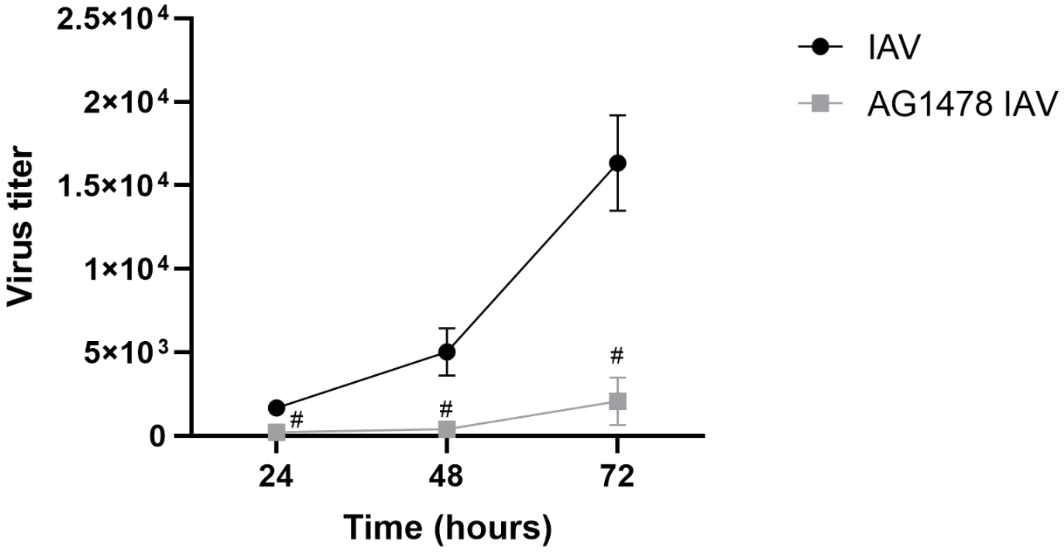


**Figure S1**. AG1478 repressed IAV production. Beas2b cells were infected with A/WSN/33 viruses at MOI=0.1. Cells were treated with 2μM AG1478 daily (direct dosing without media change). Media were collected at 24, 48 and 72 hours after infection and used for the plaque assay. Data shown are virus titer/ml, n=4, #: $p < 0.05$.

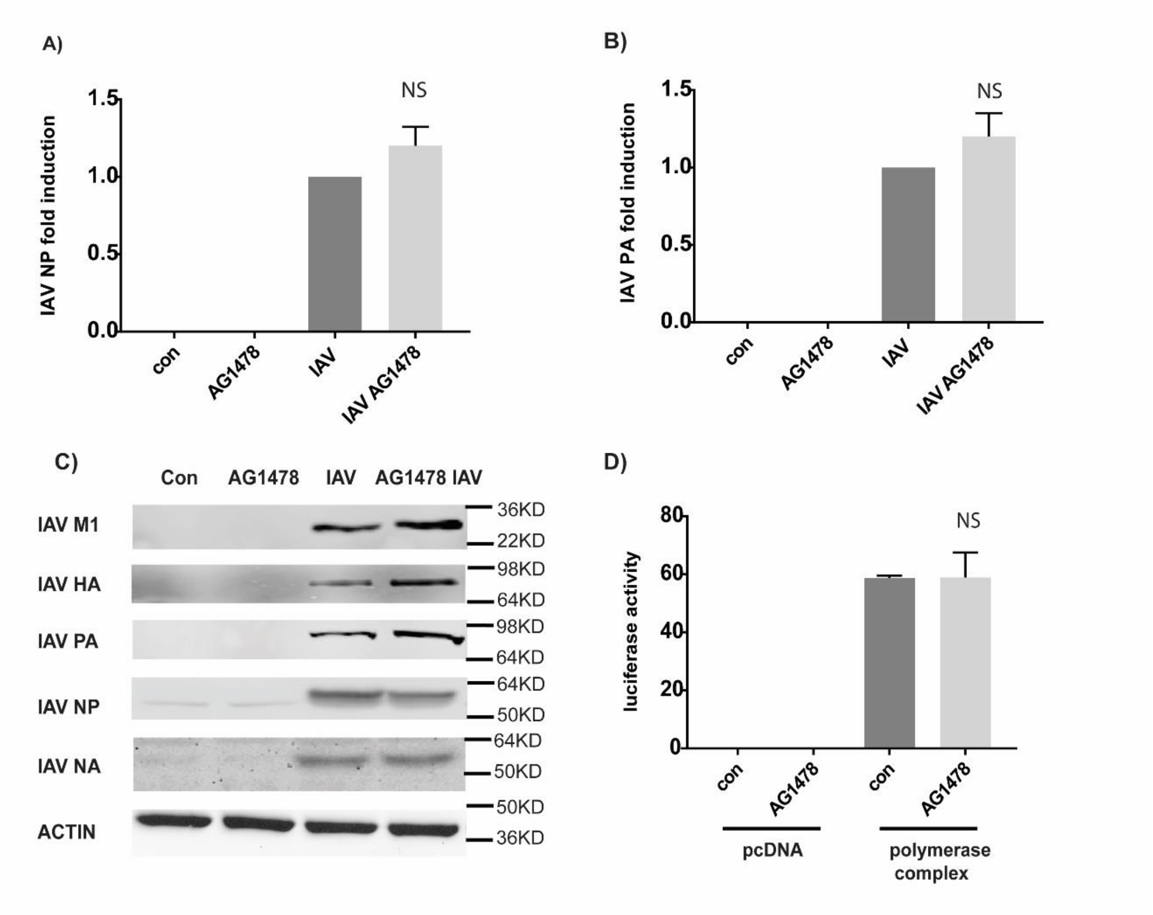


**Figure S2.** AG1478 did not affect IAV mRNA transcription, protein expression and viral polymerase activity. Beas2b cells were treated with AG1478 and infected with A/WSN/1933. A) IAV NP and B) PA gene expression were measured using real-time PCR. IAV NP forward primer: 5′- AGACTGATGGAGAACGCCAGA -3′, reverse primer 5′-TCGGTGCACATTTGGATGTAG-3′; IAV PA forward primer 5′-TCGTTC AGGCTCTTAGGGACA-3′, Reverse primer 5′AAGCAAAACCCAGGGATCATT-3′. C) IAV M1, NP, PA, HA, NA protein expression were measured using western blot analysis. ACTIN was used as a loading control. D) Minigenome assay was used to evaluate the effect of AG1478 on viral polymerase activity.

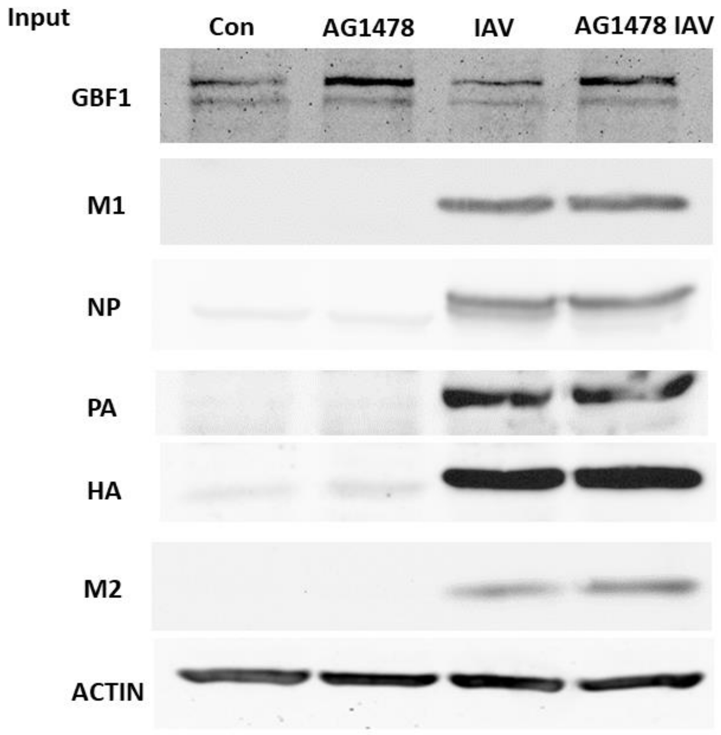


**Figure. S3.** Viral protein expression in the presence of AG1478. Beas2b cells were treated with 2μM AG1478 and infected with A/WSN/1933 for 24 hours. Total proteins, which were later used for IP analysis in Figure 6A, were analyzed for GBF1, NP, M1, PA, HA and M2. ACTIN was used as a loading control.
